# Experimental and Computational Methods for the Study of Cerebral Organoids: A Review

**DOI:** 10.3389/fnins.2019.00162

**Published:** 2019-03-05

**Authors:** Daniele Poli, Chiara Magliaro, Arti Ahluwalia

**Affiliations:** ^1^Research Center E. Piaggio, University of Pisa, Pisa, Italy; ^2^Department of Information Engineering, University of Pisa, Pisa, Italy

**Keywords:** brain, organoid, 3D culture, morphology, electrophysiology

## Abstract

Cerebral (or brain) organoids derived from human cells have enormous potential as physiologically relevant downscaled *in vitro* models of the human brain. In fact, these stem cell-derived neural aggregates resemble the three-dimensional (3D) cytoarchitectural arrangement of the brain overcoming not only the unrealistic somatic flatness but also the planar neuritic outgrowth of the two-dimensional (2D) *in vitro* cultures. Despite the growing use of cerebral organoids in scientific research, a more critical evaluation of their reliability and reproducibility in terms of cellular diversity, mature traits, and neuronal dynamics is still required. Specifically, a quantitative framework for generating and investigating these *in vitro* models of the human brain is lacking. To this end, the aim of this review is to inspire new computational and technology driven ideas for methodological improvements and novel applications of brain organoids. After an overview of the organoid generation protocols described in the literature, we review the computational models employed to assess their formation, organization and resource uptake. The experimental approaches currently provided to structurally and functionally characterize brain organoid networks for studying single neuron morphology and their connections at cellular and sub-cellular resolution are also discussed. Well-established techniques based on current/voltage clamp, optogenetics, calcium imaging, and Micro-Electrode Arrays (MEAs) are proposed for monitoring intra- and extra-cellular responses underlying neuronal dynamics and functional connections. Finally, we consider critical aspects of the established procedures and the physiological limitations of these models, suggesting how a complement of engineering tools could improve the current approaches and their applications.

## Introduction

Studies in cellular neuroscience mainly focus on *in vivo* animal models (Marbacher et al., 2018), *ex vivo* brain slices (Brai et al., 2018), and *in vitro* two-dimensional (2D) cultures (Poli et al., 2018). However, these three different experimental conditions have some limitations. Specifically, *in vivo* (animal) models, ranging from worms to non-human primates, cannot infer human cognitive abilities at the cellular level (Premack, 2007) and often fail to translate into human relevant data or clinical trials (Tsilidis et al., 2013). Brain slices, on the other hand, are very sensitive to axotomy (Humpel, 2015) and show artifacts induced by neuronal death (Dauguet et al., 2007), despite maintaining the native connections among cells. Finally, planar dissociated cultures allow the investigation of basic cellular and circuital mechanisms of neuronal networks but lack the *in vivo* microenvironment and architecture characterized by features such as neuritic outgrowth in all directions (Frega et al., 2014).

To overcome these drawbacks, recent advances in tissue engineering provide novel three-dimensional (3D) cerebral models derived from stem cells. Known as brain or cerebral organoids, these constructs mimic the 3D structure of the brain (Lancaster and Knoblich, 2014; Monzel et al., 2017; Quadrato et al., 2017) thanks to the self-organizing abilities of the stem cells they are derived from. Furthermore, human induced pluripotent stem cell (hiPSC)-derived organoids can be used to explore disease pathogenesis in a patient-oriented perspective, thus representing one of the most promising experimental models for developmental and neurodegenerative disorders (Lee et al., 2017). The scientific advances of this revolutionary technology, as well as its future prospects and limitations in modeling diseases, have been recently reviewed and discussed, exploring how these self-organized neuronal aggregates can mimic not only specific neurological and psychiatric disorders such as autism or schizophrenia (Amin and Paşca, 2018; Chuye et al., 2018; Paşca, 2018; Wang, 2018) but also the neurogenesis of congenital brain abnormalities such as microcephaly caused by the Zika virus infection during early pregnancy (Sutarjono, 2018).

Different methodological approaches, traditionally employed for characterizing brain slices and 3D *in vitro* cultures, have been applied to study brain organoids, and most investigations are geared toward assessing their reliability as novel and more accurate human brain models. In particular, advanced imaging techniques (Lancaster et al., 2013; Di Lullo and Kriegstein, 2017), delipidation protocols (Chung and Deisseroth, 2013) and novel image processing algorithms (Schmuck et al., 2017) have been proposed to observe the 3D structure of single neurons and their morphological connections at cellular and sub-cellular resolution. Well-established techniques based on current/voltage clamp (Li et al., 2017), optogenetics (Klapper et al., 2017), calcium imaging (Storm et al., 2017), and Micro-Electrode Arrays (MEAs) (Monzel et al., 2017) have been performed for monitoring intra- and extra-cellular responses underlying neuronal dynamics and functional or synaptic connections. Finally, computational models have been used to determine oxygen gradients within brain organoids (Berger et al., 2018).

**FIGURE 1:**
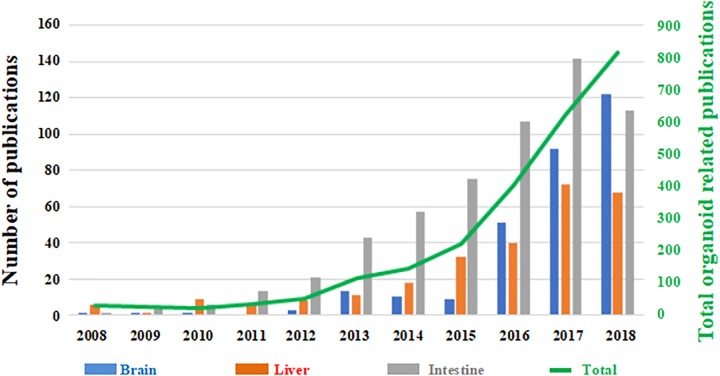
Number of papers published on organoid technology since 2008. The number of papers focused on brain organoids (blue) are shown as a function the total number of published works based on this technology (green trend line, right hand secondary axis). Papers focused on liver (red) and intestinal (gray) organoids are shown for comparison. (Source: PubMed).

However, despite the growing use of organoid technology in recent years (Figure 1), several challenges need to be addressed. In particular, a better understanding of their reproducibility in generating cellular diversity, producing mature traits and developing higher-order brain functions is still required (Quadrato et al., 2017). More in-depth knowledge and prediction of their cellular composition and distribution is also necessary. Crucially, brain organoids are often altered by non-viable centers probably due to limitations in oxygen and nutrient diffusion, thus affecting their physiological relevance and translational potential (Luo et al., 2016; Watanabe et al., 2017; Berger et al., 2018; Ogawa et al., 2018).

Neuroscientists are aware of the urgent need of improving brain organoids in terms of reproducibility and oxygen/nutrient supply as well as of fully characterizing their structural and functional features (i.e., mapping the structural and the functional connectome) for the assessment of model goodness through a quantitative comparison with their *in vivo* counterpart. In this context, we describe the state-of-art of the computational and experimental approaches recently applied to the cerebral organoids. The rationale is to inspire methodological improvements and novel applications of these brain organoids. Specifically, we first overview the organoid generation protocols commonly used in the literature (in “Brain Organoid Generation”). Then, we review the computational models employed to assess organoid formation, organization, and resource uptake (in “Computational Models”). In the sections on Structural Characterization and Integrated Electrophysiological Approaches, the experimental approaches currently provided to characterize the structure and function of cellular networks within brain organoids are discussed, focusing on methods for studying the 3D architectural of single neurons and their morphological and electrophysiological connections at cellular and subcellular resolution. Finally, in the Conclusion, we suggest how the potential of these imaging, computational and electrophysiological tools can be combined with bioprinting, fluidics and biomaterial engineering within an integrated experimental and theoretical framework so as to establish a quantitative, reproducible and accurate *in vitro* model of the human brain for a diverse range of applications.

## Brain Organoid Generation

**FIGURE 2:**
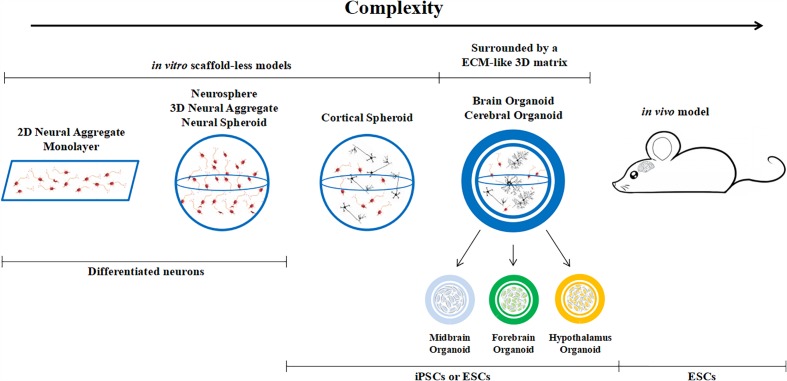
Classification of brain models, from monolayers to *in vivo* animal models.

Brain organoids are different from classical 3D cultures of neurons, which are known as neurospheres, neural spheriods or neuro-aggregates. The latter are generated from differentiated neural cells or their progenitors. The cells are usually cultured in non-adherent plates and they cluster together, growing in suspension rather than on the base of the plate. On the other hand, brain organoids originate from (usually embryonic or pluripotent) stem cells which are cultured in conditions that promote differentiation and self-organization such that the cells spontaneously develop into various brain regions resembling the developing human brain. Hence, we can define an organoid as a mini-organ (or organ sub-region) which resembles the essential, albeit immature, structural features of its upscaled counterpart (Figure 2). Brain organoids come in different sizes, ranging from 1 to 3 mm in diameter (this includes the 3D matrix, see below). The initial cell number used to generate them varies from paper to paper, but is typically between 2,500 and 10,000 stem cells (Lancaster and Knoblich, 2014; Jo et al., 2016; Monzel et al., 2017; Quadrato et al., 2017) and is a factor which certainly conditions internal nutrient and oxygen gradients.

**FIGURE 3:**
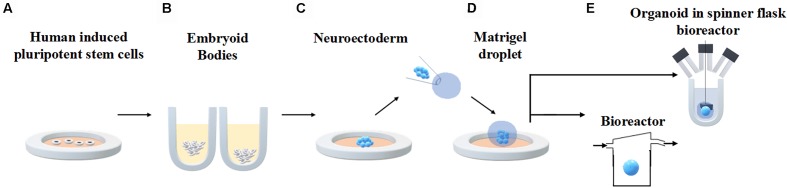
Generation of human brain organoids. Human stem cells are seeded onto plates **(A)** in order to allow embryoid body formation **(B)**. Neuroectoderm is generated after neural induction **(C)**. The cells are embedded in Matrigel droplets **(D)** and transferred to a spinner flask or a fluidic bioreactor **(E)**.

An established protocol for generating human brain organoids has been described by Lancaster et al. (2013) and Lancaster and Knoblich (2014) and shown in Figure 3. Briefly, starting from 4500 iPSC, neuroectodermal tissues are generated from embryoid bodies (EBs) through feeding with NIM (commercial Neural Induction Media) and then maintained in droplets supported by a 3D matrix composed of Matrigel, a commercially available jelly-like extracellular matrix secreted by mouse sarcoma cells. At this stage they are fed with another media cocktail for maintenance. These droplets are transferred to a spinner flask in order to enhance nutrient absorption and allow rapid tissue development, forming cerebral organoids in 10 days and defined brain regions in 30 days.

The protocol developed by Lancaster and co-workers paved the way for several other studies. Most of them observed that brain organoids become necrotic in their core at later stages of culture and healthy neurons are found only along the perimeter (Luo et al., 2016; Watanabe et al., 2017; Ogawa et al., 2018). Recently, Quadrato et al. (2017) revised this culturing protocol and facilitated the establishment of mature synapses by extending the periods of cellular growth and development. Specifically, EBs were firstly derived from about 2500 dissociated hiPSCs and subsequently transferred to intermediate induction medium after 5 days in culture. Then, NIM was added and the EBs were embedded in Matrigel and further fed with cerebral differentiation medium (CDM). Finally, brain derived neurotrophic factor was added to the medium after 30 days. The cerebral organoids obtained by performing these procedures could be cultured for up to 13 months. The expression of the H1F1-α marker demonstrated that brain organoids did not become hypoxic and levels of programmed cell death remain relatively low up to 9 months. In order to further improve the quality of brain organoids, Berger and co-workers focused on organoid culturing systems more than their generation protocols. In particular, by using fluidic devices, they not only observed a reduction of the necrotic core within organoids but also an improvement of neuronal differentiation and cellular vitality (Berger et al., 2018). The authors show that this is due to the increased flow-driven oxygen and nutrient turnover.

Although, a wide variety of chemical patterning factors have been used to drive the neuronal differentiation of specific brain sub-regions (Di Lullo and Kriegstein, 2017) such as hippocampus (Sakaguchi et al., 2015; Qian et al., 2016), midbrain (Jo et al., 2016; Monzel et al., 2017), or cerebellum (Muguruma et al., 2015), most of the literature focuses on multiple, but independent, individual brain region organoids. Using a somewhat different approach, Paşca et al. developed so-called human cortical spheroids from induced pluripotent stem cells (iPSCs) in the absence of a supporting extracellular matrix by plating the cells in non-adherent plates and supplying specific growth and patterning factors. Although, the spheroids lack the multiple brain regions and hence the organ-like quality of Lancaster’s protocol, they contain both deep and superficial cortical neurons interspersed with quiescent astrocytes and the authors claim that they remain viable for over a year (Paşca et al., 2015).

Thanks to the possibility of generating different brain regions in a dish, it is reasonable to hypothesize that the next generation of organoids will be represented by more complex *in vitro* models characterized by co-culturing “building blocks” (i.e., different brain regions) describing distinct areas of the human brain (Bagley et al., 2017). This approach would allow the observation of complex interactions such as cell migration, chemiotaxis and axon growth among different developing brain regions and enable the investigation of epilepsy and other neurological diseases. Ideally, region-specific organoids could be functionally connected in a fluidic device such as a connected culture bioreactor system which allows different tissues to communicate through a shared medium (Yin et al., 2016; Ahluwalia, 2017).

However, all of these generation protocols suffer from the so-called “batch syndrome” (Kelava and Lancaster, 2016), meaning that they show significant variability not only among the organoids from different labs or from different patients but also between organoids from the same iPSC source (e.g., karyotype). These limitations make organoids unsuitable for higher-throughput applications requiring homogeneity from well to well, such as drug screening. Besides the intrinsic sensitivity of stem cells to environmental conditions (Di Nardo et al., 2011), one of the most well-established causes of the “batch syndrome” is due to the composition of the 3D matrix (hydrogels such as Matrigel or Geltrex) whose variability is induced by the tumor materials it is derived from as well as its purification process (Kleinman and Martin, 2005). Moreover, slight differences in the thickness of the Matrigel can result in large differences in oxygen, nutrient and growth or neurotropic factor gradients in the organoid, strongly conditioning cell differentiation. Finally, heterogeneous responses over time due to cells being interrogated at different times or at different passages may also contribute to variability.

Matrigel variability could be mitigated, for example, by substituting it with chemically defined homogeneous hydrogels. In particular, synthetic biomaterials with specifically-tailored compositions could be engineered to partially reduce the intrinsic variability in cell composition (Vazin and Schaffer, 2010). This would not only improve the repeatability from batch to batch but also the cellular coating and neuronal plating over recording devices. Bioprinting also offers a technological approach to improve the reproducibility between batches. Controlled pressure and volume droplet generators (Tirella et al., 2014) could be used to modulate and control the thickness of the 3D matrix and to determine the optimum trade-off between diffusion limitations and extracellular matrix cues as well as to control the 3D organization of different cell types (Zhuang et al., 2018). Further engineering strategies could be gainfully applied to modulate the micro-environment for harnessing and controlling stem cell differentiation. Moreover, hydrogel stiffness and physiochemical properties have been already identified as key players in liver organoid formation and subsequently optimized (Takebe et al., 2013; Mattei et al., 2017). Although, assessed for stem cell technology and liver buds, the approaches can be translated and re-adapted for brain organoids to improve their reproducibility and internal core viability and to allow more control over their maturation and behavior.

## Computational Models

Organoid formation involves complex biological phenomena (e.g., stem cell differentiation into mature neurons, cell-cell contact and signaling, chemical diffusion, surface tension, and cell-substrate mechanical interactions), most of which are not well understood or easily observable (Dahl-Jensen and Grapin-Botton, 2017). Since it is well known that stem cells are highly sensitive to mechanical, biochemical and chemical stimuli (Di Nardo et al., 2011), *in silico* models provide a means to study and control single process dynamics, and thereby predict their influence on organoid growth and differentiation, providing guidance for optimizing experimental design. Moreover, in a patient-oriented perspective, computational modeling can be a powerful platform for virtual clinical trials (Karolak et al., 2018).

Unfortunately, little effort has been made in implementing models of organoid growth and differentiation, and even less for brain organoids. In particular, it has been recently demonstrated by Ahluwalia (2017), through oxygen consumption and diffusion modeling, that 3D spheroids or organoids can maintain allometric relationships between basal metabolic rate and construct mass (i.e., Kleiber’s Law, widely considered a benchmark of physiological relevance in micro-scaled *in vitro* systems). One year later, using a carefully-judged combination of image processing tools and computational models of oxygen transport and consumption, Berger et al. (2018) estimated the critical oxygen concentration (0.04 mM) necessary for ensuring cell vitality. These studies can be used as a starting point for designing cell culture systems which guarantee the threshold oxygen concentration throughout a 3D volume without exposing the organoid to a high shear stress due to media flow and ensuring that they obey Kleiber’s Law (Magliaro et al., 2019).

Using a different approach, in order to simulate the effects of cell proliferation, morphogenesis and tissue expansion occurring during organoid growth, Dahl-Jensen et al. provided a hybrid model between a cellular automata and a Douglas-Gunn diffusion scheme. Quantifying the similarity between the *in silico* and the *in vitro* outcomes, the same group demonstrated that the computational model can simulate the developing morphology of the organoid, suggesting that cell proliferation and a single inhibitory protein is enough to achieve organoid morphogenesis (Dahl-Jensen et al., 2016).

Organoids are a powerful tool for studying organogenesis. In this perspective, the evaluation of how cells move and distribute over time and arrange themselves (or spatio-temporal organization) to resemble the main architectural features of the brain allows the characterization of the mechanisms involved in brain formation such as proliferation, lineage specification and organ homeostasis. To this end, Buske et al. (2012) used computer simulations to evaluate the main structural and functional features of the organoid system as a function of the cell spatio-temporal organization. In particular they studied the possible interplay between stem and mature cells, and analyzed organoid formation in terms of cell proliferation, lineage specification and organ homeostasis (Buske et al., 2012). Comparing the results with those obtained in *in vitro* systems, they demonstrated the high sensitivity of the organoid to changes in its biomechanics, providing a framework for the selection of appropriate biomaterials for supporting organoid formation.

The combination of experimental procedures and computational models as proposed in the studies by Berger, Buske and Dahl-Jens and their respective co-workers offer several advantages for optimizing the design of more physiologically relevant *in vitro* models. Such models are also instrumental for understanding the complex mechanisms underlying organogenesis in a dish and wider collaborations between *in silico* and *in vitro* modelers should be encouraged. Finally, customized versions of the computational and theoretical frameworks established in Ahluwalia (2017) and Magliaro et al. (2019) on allometric scaling, Berger et al. (2018) on oxygen diffusion and consumption and Buske et al. (2012) on membrane biomechanics coupled with considerations on surface energy and work of cohesion, tensegrity mechanics and morphogen gradients (Dahl-Jensen et al., 2016) could be developed to predict, optimize and guide organoid formation and development.

## Structural Characterization for High-Fidelity Mapping of 3D Neuronal Structures

Cerebral organoids resemble the main structural features of the human brain recapitulating its 3D cytoarchitectural arrangement. Characterizing the three-dimensional structural organization of the cells as well as neuronal shape, size, complexity and distribution and their physical/morphological cell-cell connections within organoids is important for establishing similarities with the human brain. Quantitative and precise morphometric measures [for instance descriptors of cell size and shape such as dendrite thickness, number of dendrites, fractal number and soma sphericity (Billeci et al., 2013)] are even more crucial considering the unprecedented opportunity given by hiPSCs to provide patient-specific organoids overcoming the typical “one size fits all” experimental approach. The “one size fits all” method evaluates the average response in groups rather than individual ones, thereby neglecting some important factors deriving from the genetic profiles of individuals. Brain organoids from patient-derived hiPSCs could be used for predicting alterations in dendritic and axonal arbor associated with neuro-pathological conditions (Lancaster et al., 2013; Lee et al., 2017; Li et al., 2017; Monzel et al., 2017; Quadrato et al., 2017; Zhuang et al., 2018). Moreover, assessing the ability of these organoids to model the intricacies of the human brain and its neurogenesis can be useful for inferring how uniquely human features are managed at the cellular level (Lancaster and Knoblich, 2014; Di Lullo and Kriegstein, 2017; Lee et al., 2017; Quadrato et al., 2017). In this regard, a carefully-judged integration of advanced imaging techniques and image processing algorithms could enable high-fidelity mapping of the global neuronal organization within organoids and their morphological connections at cellular and sub-cellular scales.

Neuroanatomical features (e.g., neuron-glia connections, different cell types, and structural organization within the 3D construct) are generally extracted using optical methods such as confocal, multi-photon and light sheet microscopy, suitable for imaging within samples at cellular (sub-micrometric) resolution (Ntziachristos, 2010). Conversely, electron microscopy is commonly performed on thinner samples in order to detect the presence of structurally-defined synapses at a higher resolutions (Kelava and Lancaster, 2016; Li et al., 2017; Quadrato et al., 2017). The traditional approach for sample preparation is based on cryosections ranging from 14 to 30 μm thickness following fixation in 4% paraformaldehyde. Thicker samples may be used but are limited by the depth of penetration of light owing to optical scattering from lipids, which are present in significant amounts in the brain. Since almost all the optical imaging techniques quoted use fluorescence, the acquisition procedures are generally accompanied by fluorescent immunolabeling. Green Fluorescent Protein (GFP), as well as other biological fluorophores with different emission wavelengths can easily be integrated as genetic tags using CRISPR/Cas9 technology and are useful as markers of protein expression in transfected cells. The rationale is to reveal the presence of proteins characterizing specific cell populations (also known as molecular phenotyping) and to assess the differentiation of stem cells in mature neurons, astrocytes and oligodendrocites through the characteristic fluorescence signals of the tags (Quadrato et al., 2017; Matsui et al., 2018).

**FIGURE 4:**
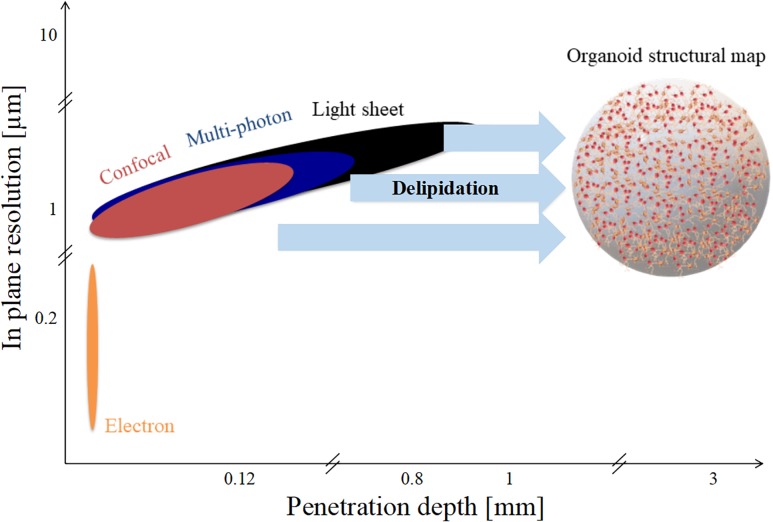
Spatial limits of modern imaging techniques applied to brain organoids. Penetration depth and in-plane resolution of specific techniques such as confocal, multi-photon, and light sheet microscopy. Delipidation increases light penetration depth but not in-plane resolution.

Organoid diameters range from 1 to 3 mm, as represented in Figure 4. However, as shown in the figure, none of the techniques mentioned have both the in-plane resolution and the depth of penetration necessary to reconstruct a high-fidelity structural connectivity map of cerebral organoids and quantitatively extract morphometrics classifying the different cell types involved (Lancaster et al., 2013; Monzel et al., 2017; Quadrato et al., 2017). Optical scattering occurs because the refractive index of lipid-rich brain tissue samples differ from that of the medium. In order to increase the depth of penetration, different optical clearing protocols have been developed in the last decade (Richardson and Lichtman, 2015). Basically tissue clearing involves exchanging the water in the sample with organic solvents, or aqueous solutions with the same refractive index as membrane lipids, such that the sample become essentially transparent. Therefore, these experimental procedures increase the depth of penetration of light and extend the depth range of optical microscopes. Clearing techniques such as CLARITY further allow permeability to both photons and macromolecules, providing sample transparency and molecular phenotyping compatibility (Chung and Deisseroth, 2013; Magliaro et al., 2016).

Although, they were originally developed for whole animal perfusion, clearing protocols can be adapted for use on vessel-free, unperfusable samples, such as brain organoids, obtaining unprecedented representations of their 3D cellular structure. For instance, Renner et al. identified internal connections between the cortical areas which appear isolated in 2D non-clarified sections using the SWITCH clearing method and subsequent in-depth confocal imaging (Renner et al., 2017). However, clearing methodologies applied to organoid technology need further optimization: in fact, a rigorous workflow for establishing the best clearing practice as well as the optimization of the immunolabeling procedure for thick samples in terms of antibody concentration and staining times are necessary to avoid much of the trial and error usually affecting these methodologies. In this regard, Magliaro and co-workers demonstrated that an optimization of tissue transparency and loss of proteins due to the clearing process itself improves both signal-to-noise and contrast-to-noise ratio during image acquisition (Magliaro et al., 2016).

**FIGURE 5:**
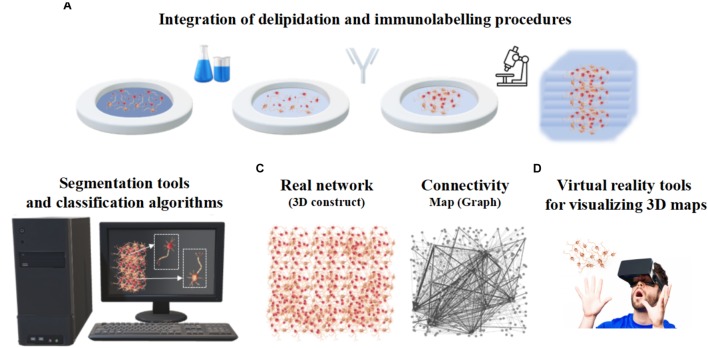
Integrated work-flow describing the three-dimensional arrangement of cells within organoids. **(A)** Integration of delipidation and immunolabeling procedures. **(B)** Segmentation tools and classification algorithms of 3D neurons based on morphometrics such as shape and size. **(C)** Structural connectivity map or graph (right) describing the three-dimensional neuronal arrangement (left). **(D)** Virtual Reality tools for visualizing 3D maps.

Despite the progress of imaging methods, the evaluation of the cellular and architectural similarities between human brain organoids and human brains (or *in vivo* animal models) are often qualitative and performed by visual inspection (Kelava and Lancaster, 2016). To the best of our knowledge, a quantitative assessment of the whole organoid micro-structure providing a detailed cell census, characterizing cell morphology and identifying cell-cell synaptic connections has not yet been performed. In particular, image processing methods are very rarely used to extract morphometric features, probably due to the lack of powerful computational algorithms and software for the automatic or semi-automatic segmentation of the neural structures (Meijering, 2010). Table 1 reports some of the attempts so far to integrate microscopy techniques with the image processing tools or software usually used for analyzing brain organoids. Since these individual procedures are not enough to characterize the whole organoid structure, it is reasonable to assume that a rigorous work-flow combining experimental protocols and computational tools will be necessary to acquire cell morphometric parameters in the future (Figure 5). In particular, integration of optimized tissue clearing protocols and novel immunolabeling procedures will allow not only better-contrasted images but also efficient and rapid thick sample staining (Magliaro et al., 2016; Figure 5A). Commercial Neurolucida (Glaser and Glaser, 1990) and freely available segmentation tools (Callara et al., 2018) may be used to extract morphometrics and quantitative shape-based neuron classification (Figure 5B), as well as high-fidelity structural maps (Figure 5C). The structural information, combined with the functional characteristics (detailed in the next section), are essential for digitalizing a computational graph of brain organoids. In the graph the nodes are the neurons and the links are the morphological or functional connections. Finally, thanks to virtual reality methods already used in neuroscience (Riddle et al., 2017; Usher et al., 2018), the visualization of 3D maps will be helpful for navigating through and interacting with the exact wiring of the neural circuits. This will be the first step towards elucidating organoid structural and functional organization (Figure 5D).

**Table 1:** Structural characterization of brain organoids using quantitative image processing: The state-of-art.

	**Methods**	**Scope**	**Application and results**	**Reference**
	
**Sub-cellular level**	Electron microscopy	Identification of sub-cellular structures	An 8 month old organoid was fixed, cut in 100 μm thick slices and acquired using backscatter electron imaging. The images were 3D rendered and manually segmented using the **VAST** lite tool, showing more axons than dendrites, appearing to preferentially run to the organoid surface.	Quadrato et al., 2017
	Confocal and multi-photon microscopy	Evaluation on cell maturation and morphology	Quantification and localization of direct contacts between the pre- and post-synaptic markers using **ImageJ.** A 3D surface reconstruction of confocal z-stacks performed with **Imaris (Bitplane)** showed an asymmetric distribution of dopaminergic neurons, unique features of the human mid-brain.	Monzel et al., 2017
**Micro and macro-anatomical level**			Organoids acquired with a confocal microscope were analyzed using **Fiji** to identify lobules staining positive for forebrain, midbrain and cerebellar/hindbrain markers and the total number of lobules were visible by DAPI staining.	Lancaster et al., 2013
			Integration of confocal microscopy analysis using **Matlab** and computational modeling for the identification of the critical oxygen concentration for cell vitality within organoids.	Berger et al., 2018
			Organoid sections imaged with confocal microscopy show neuronal layers and the formation of gaps between the organoid’s interior that resemble the ventricular spaces, evaluated using **Nikon image processing software**	Yakoub and Sadek, 2018
	Light-sheet microscopy	Evaluation of topological organization of the cells	Quantification of the surface area, overall volume and fold density in control and PTEN-mutant Hoechst-stained organoids using the **Canny Edge Detection ImageJ plugin.**	Li et al., 2017


## Brain Organoid Functions Provided by Integrated Electrophysiological Approaches

Human brain organoids can be also functionally characterized by applying specific experimental approaches able to discriminate different signal sources. The current/voltage-clamp (Li et al., 2017) and optogenetics (Klapper et al., 2017) can be used for monitoring individual cell activities, or calcium imaging for small cellular aggregations (Storm et al., 2017). Functional probing at the whole network scale can be performed in a non-invasive manner by coupling brain organoids to micro-electrode arrays (MEAs) (Monzel et al., 2017). The methodological aspects and recent applications of these approaches on brain organoids are summarized in Table 2 and discussed in this section.

**Table 2:** Electrophysiological approaches adapted to functionally characterize brain organoids: The state-of-art.

	Methods	Scope	Application and results	Reference
**Individual cells**	Current/Voltage clamp	Membrane potential and neuronal firing. Mechanistic information on ion channels.	Changes in resting membrane potentials. Cell maturation. Emergent active network (Single spikes, Burst events). Excitatory postsynaptic currents (EPSCs) Dopaminergic (mDA) neurons functionally mature. Neural development and diseases investigation. Improved long-term neuronal survival.	Hartfield et al., 2014; Paşca et al., 2015; Li et al., 2017; Di Lullo and Kriegstein, 2017; Giandomenico et al., 2018
	Optogenetics	Excitation or inhibition of the neuronal activity at high temporal and spatial resolution. Cellular polarization through light activation of specific DNA-encoded light-sensitive ion channels (i.e., optogenes) or inhibitory pumps. Cell therapy.	Modulation in real time of electrophysiological and neurochemical properties of mesencephalic dopaminergic (mesDA) neurons. Cell-type specificity, Optogene expression triggered. Broad diversity of cellular responses.	Steinbeck et al., 2015; Rajasethupathy et al., 2016; Klapper et al., 2017; Quadrato et al., 2017
**Small cellular aggregations**	Calcium imaging	Characterization of the Ca^2+^ status and changes in fluorescence induced by the binding of the Ca^2+^ ions with genetically encoded calcium indicators or small molecules based on the aminopolyearbowlie acid BAPTA	Homogeneous fluorescence induced by calcium detection reagents such as Fluo-4 direct. Emergence of spontaneous and single cell tracings of calcium induced by glutamate and TTX application.	Lancaster et al., 2013
**Network scale**	Micro-electrode array (MEA)	Characterization of the extracellular electrophysiology. Acquisition of long-term spontaneous recordings and evoked responses induced by chemical or electrical stimulation at 60 or 120 up to 4,000 or 10,000 electrodes	Mono- and biphasic spikes closely in time. Firing frequency reduction induced by chemical perturbation (quinpirole treatment) on midbrain dopaminergic neurons (mDNs) Neuronal dynamics from spontaneous activity.	Monzel et al., 2017; Giandomenico et al., 2018


**FIGURE 6:**
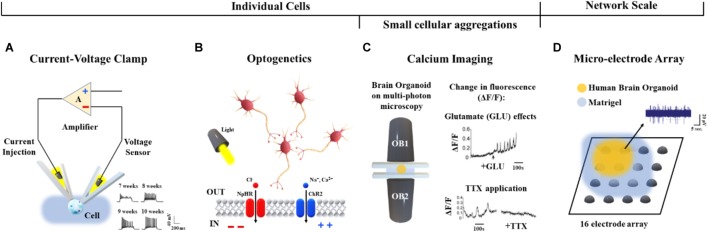
Functional characterization approaches applied to human brain organoids. **(A)** Voltage/current clamp technique and representative recordings (bottom, right) from hiPSC-derived organoids after 7, 8, 9, and 10 weeks *in vitro* (Hartfield et al., 2014). **(B)** Cellular polarization changes can be achieved in a cell-type-specific manner via optogenetics by means of light activation of specific DNA-encoded light-sensitive ion channels (e.g., the channel rhodopsin ChR2 colored in blue) or inhibitory pumps (e.g., the halorhodopsin NpHR colored in red) (Rajasethupathy et al., 2016). **(C)** Calcium imaging based on multi-photon microscopy (left) and detected changes in fluorescence (ΔF/F) induced by Glutamate (top, right) and TTX (bottom, right) (Lancaster et al., 2013). **(D)** Representative midbrain organoid coupled to a 16-electrode array in a 48-well tissue culture plate and spontaneous activity from one active recording site.

Briefly, current/voltage-clamp (Figure 6A) is commonly used to investigate individual cellular activity and provide mechanistic information on ion channels (Cummins et al., 2009). This technique, applied to brain organoids, allows the detection of emergent active networks producing complex synaptic events associated with postsynaptic neuronal spike firing (Paşca et al., 2015). Changes in resting membrane potentials (Hartfield et al., 2014), functionally active midbrain dopaminergic neurons (Jo et al., 2016), and cell maturation (Di Lullo and Kriegstein, 2017) also have been observed. These functional aspects, as well as the excitatory/inhibitory postsynaptic currents, the neuromelanin-like granules structurally similar to those isolated from human *substantia nigra* tissues and the inactivating inward/outward currents, support the reliability of cerebral organoids for modeling human brain (Paşca et al., 2015; Jo et al., 2016; Li et al., 2017). Furthermore, recent whole-cell voltage clamp recordings of individual neurons from air-liquid interface cerebral organoids show improved long-term survival of the cells distributed in three-dimensional space (Giandomenico et al., 2018).

As shown in Rajasethupathy et al. (2016), regional targeting capability without neuron-type specificity has been additionally provided by applying local electrical stimuli as well as rapidly changing magnetic fields to organoids. Conversely, optogenetic approaches (Figure 6B) based on light activation of inhibitory pumps or specific DNA-encoded light-sensitive ion channels (i.e., optogenes; Deisseroth and Schnitzer, 2013) have been adapted to human brain organoids in order to target specific cell-types and establish a consolidated methodology for investigating cellular excitability at high temporal and spatial resolution (Klapper et al., 2017). In particular, the cellular polarization changes induced by the light stimulation of photoreceptor-like cells were shown to excite and inhibit neuronal activity within brain organoids, offering an opportunity for studying aspects of the regional complexity, cellular diversity and circuit functionality of the brain (Quadrato et al., 2017). Furthermore, light-modulated electrophysiological and neurochemical properties of mesencephalic dopaminergic neurons within human embryonic stem cell-derived organoids also provide an important contribution in cell therapy driving real-time recovery from lesion-induced Parkinsonian motor deficits (Steinbeck et al., 2015).

Current/voltage-clamp and optogenetic methods can be used to investigate the neuronal dynamics involved at the single cell level. In order to functionally characterize neural aggregates, Calcium (Ca^2+^) imaging has been applied to the human brain organoids (Figure 6C). Studies based on confocal or multi-photon microscopy (Grienberger and Konnerth, 2012) showed fluorescence changes induced by the binding of Ca^2+^ ions with genetically encoded calcium indicators or small molecules based on the aminopolycarboxylic acid BAPTA (Romoser et al., 1997). Calcium imaging based methods adapted to live human brain organoids were also reported by Lancaster et al. (2013).

Although, calcium imaging allows functional characterization of single neurons and small aggregates, this approach does not provide a more complex analysis of the dynamics involved at the whole network scale. Technologies such as MEAs may be used to acquire the electrophysiology from multiple recording sites, as well as to record cell responses evoked by chemical (Pancrazio et al., 2003) or electrical (Wagenaar et al., 2004) perturbations. In particular, MEAs simultaneously monitor long-term spontaneous recordings and responses induced by stimulation protocols using from 60 or 120 (Poli et al., 2018) up to 4,000 or 10,000 electrodes (Berdondini et al., 2009). Therefore, this technology allows high control of the system, supporting a direct reconstruction of the underlying functions and dynamics at the network scale (Poli et al., 2017). To the best of our knowledge, only a few reports describing the functional connectivity of brain organoids coupled to planar MEAs have been published. For example, Monzel et al. (2017) as well as Giandomenico et al. (2018) recently derived human midbrain organoids and plated them over an integrated MEA system for recording (Table 2 and Figure 6D). In the future, cerebral organoids could be also coupled to three-dimensional MEAs (Musick et al., 2009; Yang et al., 2016) which better conform to their shape - for monitoring spontaneous or evoked neuronal signals from multiple layers.

Previous studies focusing on complex networks at the whole-brain scale of human neuroimaging (Bullmore and Sporns, 2009) and at a cellular scale in animal models (Maccione et al., 2012) suggest a strong interplay between the synaptic connections and neuronal morphology and the underlying electrophysiological dynamics. Therefore, a multi-disciplinary approach integrating these electrophysiological methods with morphological architectures obtained from structural characterization could be used to generate more realistic and refined functional networks coherent with and possibly superposed on the network structure (Ullo et al., 2014).

## Conclusion

**FIGURE 7:**
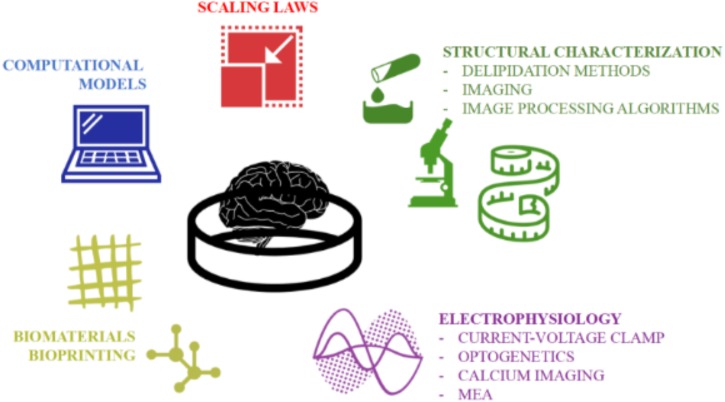
The multidisciplinary tools toward establishing a quantitative and accurate *in vitro* model of the human brain. An integration of computational and experimental approaches would allow a rigorous structural and functional characterization of the neuronal networks within organoids and their validation as physiologically relevant downscaled *in vitro* models of the human brain.

The development of stem cell-based organoids is one of the most fascinating and promising techniques for providing a physiologically relevant downscaled *in vitro* model of the human brain. However, since this technology is relatively young (Lancaster et al., 2013), the generation protocols and characterization procedures still need refinement. Firstly, the intrinsic stochasticity and sensitivity of the stem cells to their microenvironment contribute to the heterogeneity of organoids (i.e., the “batch-syndrome”). In addition the variability of the hydrogel matrix (i.e., Matrigel or Geltrex), a necessary feature of current brain organoid generation protocols, doubtless influences their reproducibility. Second, brain organoids (particularly those generated from ≥ 4,000 stem cells) often suffer from oxygen and nutrient deprivation due to transport limitations (Berger et al., 2018). As a result, they are often reported to possess necrotic cores which likely affect not only their morphology but also their functional behavior. Therefore, a new reproducible and standardized production pipeline -preferably supported by computational and characterization tools- is deemed necessary to maintain self-organizing complexity and cell vitality. However, at present, bespoke computational tools supporting biologists in optimizing organoid generation and characterizing their structure and functions are still lacking. Efforts should be made to develop novel computational models and experimental procedures for better investigating the network dynamics as well as more complex cell-cell connections (e.g., synapses) and cell-microenvironment interactions (e.g., hypoxia). In this direction, we suggest an *ad hoc* work-flow overcoming the aforementioned limitations by combining the functional methodologies and imaging techniques illustrated in Figure 7 for better assessing the ability of cerebral organoids to model specific pathological and developmental processes in the human brain. In order to support the realization of this integrated approach, we have described and discussed the well-established procedures used for generating organoids, as well as the current computational and experimental techniques used for simulating and measuring their structural and functional organization as they differentiate and mature. We hope that this review inspires new computational and technology driven ideas for methodological improvements and novel applications of brain organoids.

## Author Contributions

All authors contributed to the preparation and editing of the manuscript.

## Conflict of Interest Statement

The authors declare that the research was conducted in the absence of any commercial or financial relationships that could be construed as a potential conflict of interest.
